# Dihydroartemisinin as a pharmacokinetically constrained immune-conditioning pulse in cancer therapy

**DOI:** 10.3389/fimmu.2026.1955672

**Published:** 2026-10-29

**Authors:** Pei Wei, Jiahua Wang, Yuan Cao, Yongxiang Song, Zhiyong Wang

**Affiliations:** 1 Department of Immunology, Zhuhai Campus of Zunyi Medical University, Zhuhai, China; 2 Shengli Clinical Medical College of Fujian Medical University, Fujian, China; 3 Department of Thoracic Surgery, Affiliated Hospital of Zunyi Medical University, Zunyi, China

**Keywords:** dihydroartemisinin, immune relay, immunomodulation, pharmacokinetics, tumor microenvironment

## Abstract

Mechanistic assignment in cancer immunology often assumes that durable antitumor effects reflect sustained pharmacologic pressure on tumor cells. Dihydroartemisinin (DHA) provides a useful test case for revisiting this assumption because clinically achievable systemic exposure is brief and free-drug availability is constrained by substantial protein binding. We therefore propose that brief systemic DHA exposure may act as a pharmacokinetically constrained immune-conditioning pulse whose durable antitumor effects, when present, could involve persistent tumor-intrinsic injury, immune relay, or both. Within this framework, transient tumor or stromal stress may be converted into longer-lived antitumor activity through myeloid-state transition, dendritic-cell activation, relief of immune exclusion, and subsequent CD8^+^ T-cell priming. Current evidence does not yet establish a dominant immune mechanism under exposure-matched conditions. Suppressive myeloid-state change, a tumor stress–dendritic cell–CD8^+^ T-cell axis, and direct lymphocyte responsiveness are competing hypotheses for exposure-matched testing alongside direct tumor-cell effects. This framework informs exposure-matched experimental design, biomarker development, and rational immunotherapy combinations, and provides a falsifiable prioritization scheme based on exposure matching, temporal precedence, persistence after washout, and *in vivo* necessity.

## Introduction

In cancer immunology, drug exposure and antitumor benefit often operate on different timescales (1). Continuous target engagement may be necessary for some therapies, particularly when activity depends on intracellular occupancy in tumor cells. Other treatments may need only a brief initiating event if the resulting signal is amplified, transmitted through the tissue, or retained by the tumor immune microenvironment (2). Danger signaling, myeloid reprogramming, dendritic-cell activation, antigen transport, and adaptive immune priming can all persist for days or weeks after the original stimulus has ended (3, 4). The duration of drug exposure alone may therefore be a poor guide to the duration of biological activity.

Dihydroartemisinin (DHA) and related artemisinin compounds have been studied extensively in cancer models. Preclinical studies describe oxidative stress, apoptosis, ferroptosis-associated changes, and inhibition of tumor growth, while recent reviews discuss the prospects for translation and combination therapy (5). A newer line of work has broadened this picture. DHA has been associated with immunogenic tumor stress, activation of dendritic cells, contraction of suppressive myeloid populations, increased CD8^+^ T-cell activity, and improved responses to immunotherapy (6, 7).

The unresolved issue is therefore not whether DHA can affect tumors, but how its dominant *in vivo* mechanism should be assigned under clinically relevant exposure conditions. The field has been shaped by an implicit default narrative: when DHA suppresses proliferation or induces death under prolonged high-concentration *in vitro* exposure, this is often taken as the preferred explanation for *in vivo* efficacy. The issue is not that direct tumor-cell effects are irrelevant, but whether their relative contribution can be established from experiments that do not resolve local exposure or persistent post-exposure injury (8, 9). This creates a recurring mechanism-assignment bias in which experimentally convenient cytotoxic exposure regimes come to dominate *in vivo* interpretation even when the relevant systemic exposure architecture is brief.

We therefore propose a shift in emphasis. Direct tumor-cell effects and immune relay should not be considered mutually exclusive mechanisms. A brief DHA exposure may induce tumor-cell stress, sublethal dysfunction, delayed cell death, or other tumor-intrinsic consequences that persist after extracellular drug clearance (3, 4, 10, 11). These consequences may contribute directly to tumor control while also generating antigens, danger signals, chemokine changes, or altered phagocytic cues that initiate or amplify immune relay (3, 4, 10, 11). The relevant mechanistic distinction is therefore not between tumor-cell effects and immune-mediated effects, but between effects that require continued pharmacological exposure and effects that persist or are relayed after a brief pulse.

## Pharmacokinetic exposure architecture as a boundary for immune-mechanism assignment

Any serious discussion of DHA mechanism in cancer immunity should begin with its exposure architecture in humans (9, 12). Available *in vivo* protein-binding data indicate that only a limited fraction of circulating DHA is unbound (9, 13). In one study, the mean unbound fraction of DHA in human plasma was approximately 0.07–0.12, depending on the population studied, implying that total plasma concentrations overestimate pharmacologically available free-drug exposure (13).

The clinical pharmacokinetics of DHA favor a brief-input interpretation. Reviews of artesunate and DHA show rapid conversion of artesunate to DHA after intravenous, intramuscular, oral, or rectal administration, followed by rapid loss of circulating DHA (12). Here, systemic DHA exposure refers to DHA measured after administration of DHA itself or formation from artesunate; the administered drug, route, formulation, and parent-drug and metabolite profiles should be specified rather than treated as interchangeable (9, 12, 14, 15). In patients with metastatic breast cancer receiving long-term oral artesunate, salivary DHA tracked total plasma DHA with a slope of 0.116, close to reported estimates of the unbound plasma fraction (9, 13). Saliva does not reproduce tumor interstitial exposure, so this observation cannot establish intratumoral concentrations. It does support the narrower conclusion that freely available drug is substantially lower than total circulating drug. A parenteral artesunate study in healthy volunteers and a population analysis of DHA–piperaquine treatment in malaria likewise describe short systemic persistence of DHA, but involve different drug sources and dosing contexts (14, 15).

Total plasma concentration, unbound plasma concentration, tumor-interstitial exposure, intracellular exposure, and pharmacologically active exposure are related but non-equivalent quantities. Intracellular drug abundance does not itself establish active exposure, which also depends on drug activation and concentration–effect relationships in the relevant compartment. Available human data principally constrain systemic plasma exposure and do not establish the magnitude, duration, or spatial distribution of unbound DHA within tumors (9, 12–15). Tissue partitioning, local protein or lipid binding, cellular uptake, stromal sequestration, intracellular distribution, local metabolism, and slow tumor washout could potentially increase or prolong local exposure relative to that predicted from plasma data.

The consequence is a requirement for exposure-resolved comparison, not a change in mechanistic priority based on plasma PK alone. After a transient systemic exposure, candidate mechanisms include sustained local drug action, persistent tumor-intrinsic injury, direct immune-cell responses, and immune relay. Prolonged free-drug exposure in the tumor, selective retention, and other exposure-extending processes require verification rather than being assumed or excluded. Direct cytotoxicity remains possible under standard systemic dosing. The unresolved issue is whether it can account for the dominant *in vivo* effect within these exposure limits.

On a nominal basis, commonly used *in vitro* DHA concentrations are substantially higher and are maintained substantially longer than the estimated free systemic exposure (8, 12, 13, 16). However, nominal concentrations added to culture medium are not equivalent to unbound concentrations in the culture system, and estimated unbound plasma concentrations do not directly define unbound exposure in the tumor microenvironment. This comparison should therefore be interpreted as a qualitative concentration–time discrepancy and as a rationale for exposure-matched testing, rather than as a direct quantitative comparison or as evidence for a definitive concentration threshold below which direct tumor-cell effects cannot occur *in vivo*.

Tumor-cell killing *in vitro* is often studied with 10–100 μM DHA maintained for 24–72 hours (16), whereas selected clinical studies of DHA exposure following systemic artesunate administration have reported total plasma DHA peaks in the approximate range of 1–3 μM (8, 12). Applying the reported unbound fractions of 0.07–0.12 yields an approximate free-plasma peak estimate of 0.07–0.36 μM (13); this estimate should not be interpreted as a surrogate for unbound tumor-interstitial or intracellular exposure. The *in vitro* values are nominal concentrations added to culture medium, whereas the latter is an estimate of unbound plasma exposure and does not account for tumor distribution, local binding, intracellular uptake, or tissue retention. Accordingly, these data indicate a qualitative concentration–time mismatch and support the need for exposure-matched experiments, but they do not establish a quantitative threshold that excludes direct tumor-cell effects *in vivo*. A brief DHA exposure may still produce persistent tumor-intrinsic injury after drug clearance.

Human plasma pharmacokinetics therefore provide a boundary condition rather than a complete description of tumor exposure. These data justify caution when prolonged, high-nominal-concentration cell-culture experiments are used to assign a dominant *in vivo* mechanism, but they do not exclude transient direct tumor-cell effects, persistent tumor-intrinsic injury after a brief exposure, or prolonged local exposure in particular tumor settings.

Clinically relevant experiments do not have to reproduce the human concentration–time curve point by point. They should nevertheless report total and unbound AUC, Cmax, pulse length, dosing interval, local retention, and, where an active concentration is established, time above that concentration, with the drug source and route specified. Concentration–effect measurements should assess possible PK/PD hysteresis, because delayed effects may reflect retained active drug, persistent tumor-intrinsic injury, or immune-state change. A response that survives washout supports biological persistence only when residual active exposure is assessed, and does not by itself identify immune relay. Single pulses, repeated intermittent pulses, continuous exposure, and sustained-release or local delivery should be compared as dist*inct exposure archit*ectures.

## From short pharmacologic input to immune relay

Once DHA is treated as a transient input, the central mechanistic question is how tumor and immune compartments carry its effects forward. Demonstrating injury while DHA is present does not distinguish sustained drug action, persistent tumor-intrinsic injury, and immune relay. We use immune-conditioning pulse operationally for a transient DHA exposure hypothesized to initiate an immune-relevant biological change that persists after active drug exposure has ended; the initiating compartment need not be an immune cell. We use immune relay for the proposed transmission or amplification of that event through tumor and immune compartments (**Figure 1**).

**Figure 1:**
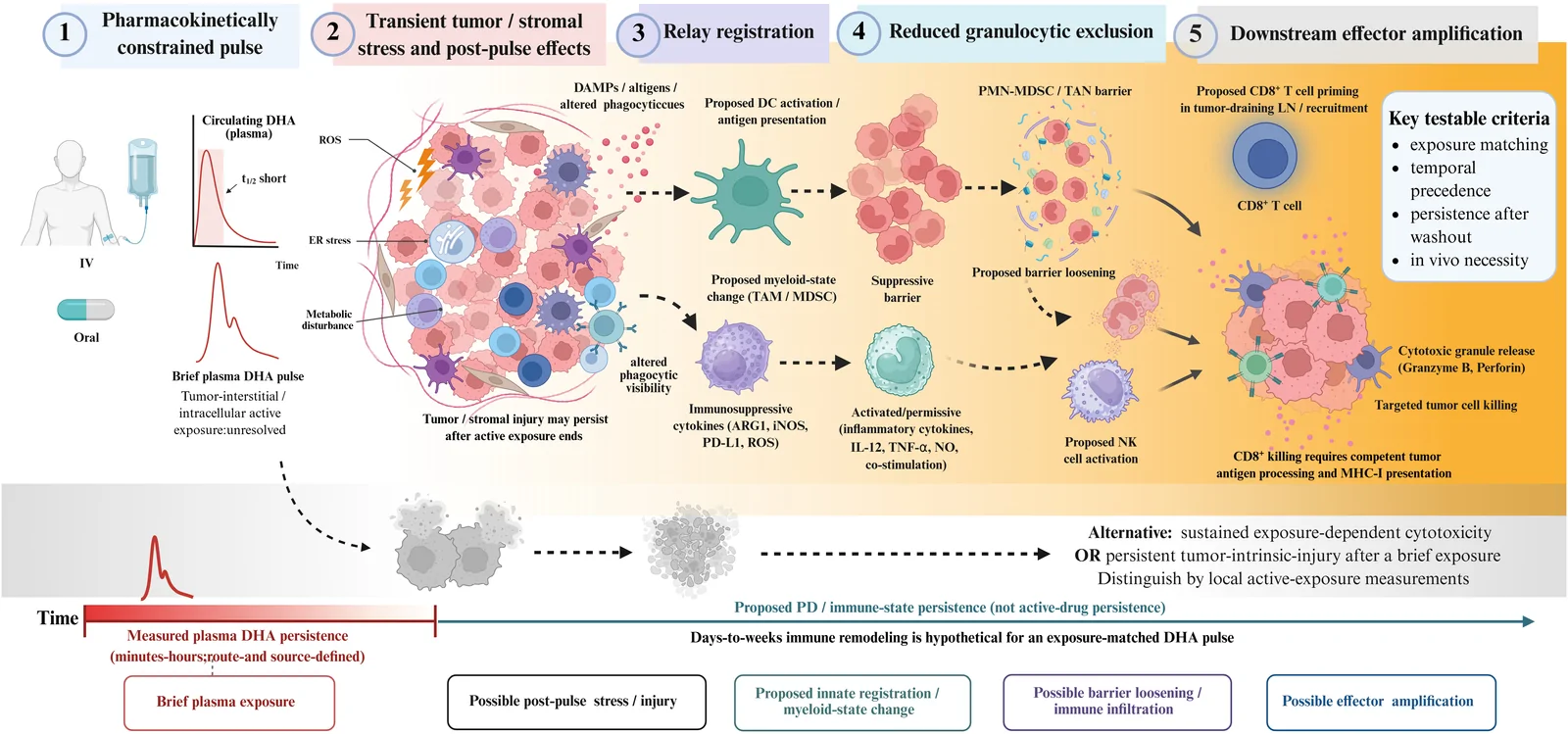
A testable pulse-triggered immune-relay model for DHA in the tumor immune microenvironment. (1) Pharmacokinetically constrained pulse: rapid loss of circulating DHA following route- and source-defined administration yields a brief plasma exposure; tumor-interstitial and intracellular active exposure remain to be resolved. (2) Transient tumor/stromal stress and post-pulse effects: the short input may induce ROS, ER stress, metabolic disturbance, and tumor-cell injury that could persist after active drug exposure has ended. (3) Relay registration: tumor-cell stress or injury may provide DAMPs, antigens, and altered phagocytic cues for DC activation and myeloid-state change (TAM/MDSC). (4) Reduced granulocytic exclusion: myeloid-state change could loosen PMN-MDSC/TAN barriers and permit immune infiltration. (5) Downstream effector amplification: relayed signals could support CD8^+^ T-cell priming in the tumor-draining lymph node, recruitment, and NK-cell activation; CD8^+^ T-cell killing requires competent tumor antigen processing and MHC-I presentation, while direct lymphocyte responses remain an alternative. The lower gray pathway represents sustained exposure-dependent cytotoxicity or persistent tumor-intrinsic injury after a brief exposure, to be distinguished by local active-exposure measurements. Bottom timeline: separates measured plasma drug persistence from proposed pharmacodynamic and immune-state persistence; days-to-weeks immune remodeling is hypothetical for an exposure-matched DHA pulse. Dashed mechanistic arrows denote proposed DHA-specific relationships, not an established temporal sequence; solid arrows denote only established general immune processes, and colors do not rank mechanisms. Inset box: four key criteria for mechanism assignment. Created in BioRender. Wang, J. (2026) https://BioRender.com/abqe6fi.

A brief exposure may have lasting effects through pathways with their own capacity for persistence. Transcriptional reprogramming, antigen presentation, cytokine remodeling, altered phagocytic recognition, and changes in the spatial access of immune effectors can all extend the consequences of a transient trigger. Under this model, pharmacokinetic persistence means continued active drug exposure, pharmacodynamic persistence means continued biological effects after that exposure has ended, and immune-state persistence means continued changes in immune composition or function. Repeated intermittent pulses may accumulate pharmacodynamic or immune-state changes without continuous drug exposure.

Tumor-cell stress may initiate the sequence, and its biological consequences may persist even when extracellular DHA is no longer detectable. A brief exposure could commit a subset of tumor cells to delayed death, sustain oxidative or mitochondrial dysfunction, or alter phagocytic visibility after washout (17–19). These tumor-intrinsic consequences may contribute directly to tumor control while also producing antigens, DAMPs, chemokine changes, or altered phagocytic cues that are subsequently registered by myeloid and dendritic-cell compartments (3, 4, 20–22).

Recent work on DHA supports this reframing but does not yet prove it. In hepatocellular carcinoma models, DHA induced immunogenic cell death features, activated dendritic cells, increased intratumoral CD8^+^ T cells, reduced immunosuppressive MDSCs, and enhanced anti-PD-1 and CAR-T efficacy (6, 10). Combined with cisplatin, DHA restored tumor immunogenicity, increased DAMPs release and dendritic-cell phagocytosis, and strengthened anticancer immunosurveillance through PERK/eIF2α signaling (17). Engineered DHA platforms and derivatives designed for mitochondrial targeting or iron-assisted delivery have likewise shown that intensified oxidative stress can strengthen immunogenic cell death-like responses and sensitize tumors to checkpoint inhibition (18, 19). DHA has also been associated with microbiome-mediated sensitization to anti-PD-1 treatment in hepatocellular carcinoma (22).

These studies provide important support for the biological plausibility of DHA-associated immune modulation, but their pharmacokinetic relevance to route- and source-defined systemic DHA exposure remains uncertain. Several studies used prolonged or continuous exposure, nominal concentrations whose relationship to clinically relevant free-drug levels was not established, combination treatment, chemically modified DHA derivatives, or engineered delivery systems that may alter local concentration, tissue distribution, subcellular localization, or exposure duration. Such studies demonstrate that DHA-related tumor stress can engage immune-relevant pathways, but they do not by themselves establish that these pathways dominate DHA-associated antitumor effects under route- and source-defined, exposure-matched conditions.

Overall, these findings do not define a universal DHA mechanism across tumor types, formulations, or therapeutic settings. Their narrower implication is that DHA-associated tumor stress may be recognized by the tumor immune microenvironment and may alter the response to immunotherapy. This supports testing an immune-relay model without establishing its operation after a single brief exposure.

Evidence for immune relay should therefore be treated as suggestive rather than conclusive. Establishing its *in vivo* relevance under pharmacokinetically representative conditions is a central experimental aim of the framework proposed here.

## Candidate initiating compartments under pharmacokinetic constraints

In this framework, “PK-compatible” denotes temporal plausibility under brief systemic exposure. It does not indicate that a mechanism has been directly demonstrated under exposure-matched conditions, that the relevant compartment is the earliest or primary receiver of DHA, or that the pathway is necessary for therapeutic efficacy *in vivo*. Accordingly, the mechanisms discussed below are presented as competing hypotheses for testing rather than as an established universal immune hierarchy.

## Suppressive myeloid compartments as candidate receivers of the DHA pulse

Among the pathways that could connect a brief pharmacologic input to a durable biological output, suppressive myeloid compartments are candidate receivers to examine alongside tumor cells, dendritic cells, and lymphocytes. Tumor-associated macrophages, monocyte-derived myeloid cells, and MDSC populations are well placed to determine whether transient tumor stress is resolved tolerogenically or converted into productive antitumor immunity (23–26). They influence antigen presentation, inflammatory tone, phagocytosis, chemokine production, and the threshold at which T-cell responses are suppressed (23–26).

From a cancer immunology perspective, suppressive myeloid compartments are attractive not simply because they are abundant, but because they gate whether local injury becomes immunologically silent or immunologically productive. Early shifts in suppressive myeloid state could propagate into reduced inhibitory signaling, improved dendritic-cell support, altered cytokine and chemokine landscapes, relief of immune exclusion, and increased sensitivity to checkpoint-based therapies (23–25, 27). Accordingly, myeloid-state change is a candidate initiating event or downstream response. Depletion denotes cell loss, altered recruitment denotes changed cell influx, phenotypic change denotes altered markers, transcriptional reprogramming denotes altered gene-expression programs, and functional reprogramming requires evidence of changed activity; these outcomes should not be inferred from one another.

DHA-associated reductions in immunosuppressive MDSCs support this possibility but do not distinguish depletion from altered recruitment or establish functional reprogramming (6). A DHA study reported TAM phenotypic and functional changes after 18-hour exposure, while a 2025 artesunate study linked STAT1/IRF1-associated TAM changes to enhanced phagocytosis, antigen cross-presentation, and T-cell activity (28, 29). In contrast, artesunate inhibited M1 polarization in a sepsis-associated liver-injury model, indicating that inflammatory activation is not a uniform response across settings (30). Even so, the field still lacks sufficiently exposure-matched and compartment-resolved studies to determine whether these myeloid changes are a dominant single-agent mechanism, a combination-dependent amplifier, or a context-specific contributor. Resolving this distinction will require early time-point studies that ask whether myeloid-state change precedes later CD8^+^ T-cell influx, rather than inferring mechanism from late endpoint immune composition alone.

## A transient tumor stress–dendritic cell relay–CD8^+^ T-cell priming axis

Another possibility is that transient tumor stress is relayed by dendritic cells into CD8^+^ T-cell priming. This mechanism could fit a short DHA exposure window because cross-presentation need not require sustained drug exposure (20). The initiating exposure need only produce sufficient immunogenic stress to make danger signals or tumor antigens available to dendritic cells (3, 4).

The necessary immunologic processes are well established. Dendritic cells cross-present tumor antigens and prime tumor-reactive CD8^+^ T cells. In the proposed relay, cDC1 could acquire antigens from stressed or dying tumor cells and transport them to the tumor-draining lymph node, where migratory or antigen-receiving resident cDC1 could cross-present them and prime CD8^+^ T cells (20, 21). Effective anti-PD-1 treatment partly relies on continued T cell–dendritic cell crosstalk mediated by IFN-γ and IL-12 (31). Calreticulin exposure, ATP release, and HMGB1 release further explain how stressed or dying tumor cells may become visible to the immune system (3, 4, 32). The contribution proposed here is not discovery of this relay, but exposure-matched testing of whether its temporal sequence, persistence, and causal necessity explain DHA-associated efficacy.

Transient tumor stress may thus be consequential when dendritic cells convert it into a durable adaptive response. If cDC1-dependent priming is required, Batf3 deficiency or other disruption of cDC1 function should attenuate DHA-associated tumor control (33). Existing DHA-specific evidence supports the plausibility of this experiment, but should not yet be interpreted as proof that the relay dominates across all settings.

## PMN-MDSC and tumor-associated neutrophil barriers as emerging upstream constraints

The PMN-MDSC/tumor-associated neutrophil axis is another candidate upstream constraint because granulocytic populations can suppress immune function and restrict effector-cell access. Neutrophil-like cells occupy a spectrum ranging from antitumor N1-like to protumor N2-like states (34). Distinct PMN-MDSC populations have been described in patients and tumor-bearing models, and these populations can suppress NK cells and broader antitumor immunity (35, 36).

Within a PK-constrained framework, weakening an upstream granulocytic barrier could allow a brief drug pulse to yield durable immunologic effects by improving T-cell access rather than sustaining direct tumor-cell injury. Recent data suggest DHA may modulate neutrophil states: a 2026 study showed DHA promoted N1-like changes in differentiated NB4 neutrophil-like cells and increased their activity against hepatocellular carcinoma cells in co-culture (37). Even so, this axis remains an emerging hypothesis, not an established dominant pathway. Its conceptual value lies in the notion that short pharmacologic inputs can have durable effects by reshaping immune exclusion.

## CD8^+^ T cells and NK cells as candidate direct receivers and relay effectors

CD8^+^ T cells and natural killer cells are essential antitumor effectors (38, 39). Their roles as direct receivers, downstream effectors, or amplifiers remain competing possibilities under exposure-matched conditions. The distinction matters because greater cytotoxic-lymphocyte abundance after treatment is often interpreted mechanistically, even though it may be a downstream consequence of earlier changes in myeloid suppression, dendritic-cell activity, antigen presentation, or immune exclusion (20, 40, 41).

In many tumor models, stronger CD8^+^ T-cell or NK-cell activity may follow upstream changes in antigen availability, chemokine gradients, suppressive myeloid activity, or granulocytic barriers (24, 40, 41). Increased CD8^+^ T-cell infiltration after DHA treatment should therefore not, by itself, be taken to demonstrate a direct action of DHA on T cells without compartment-resolved evidence separating direct lymphocyte responses from upstream relay. Similarly, increased NK-cell output may result from release of PMN-MDSC-mediated suppression rather than direct sensing of the DHA pulse by NK cells (24).

Direct lymphocyte responsiveness should be tested using the same exposure and causal criteria as tumor-intrinsic and relay mechanisms. Brief *ex vivo* exposure of purified CD8^+^ T cells or NK cells could distinguish effects on activation, survival, differentiation, or function, while compartment-specific perturbations could assess their contribution to *in vivo* efficacy. Artesunate-associated T-cell activation in lung-cancer models and DHA-associated inhibition of activation markers in HIV-donor PBMC cultures illustrate different response directions; the latter used 100–1000 μM DHA for 48 hours and neither context establishes direct lymphocyte responsiveness to a clinically representative DHA pulse (29, 42).

These considerations support a pharmacokinetically constrained framework for immune-mechanism prioritization in which candidate pathways are compared by biological plausibility, compartment-specific exposure, temporal sequence, and the current strength of supporting evidence without a default ranking (**Table 1**). Importantly, this framework is intended to guide testing rather than to declare a final universal mechanism.

**Table 1:** Immune-mechanism prioritization for DHA under pharmacokinetically constrained systemic exposure.

Candidate mechanism	Putative initiating compartment	Exposure requirement to test	Current evidence strength	Implication for immune-mechanism assignment
Sustained direct tumor-cell action or persistent post-pulse injury	Tumor cells	Local active exposure or persistent injury after a brief pulse; unresolved	Strong in conventional continuous-exposure *in vitro* systems; contribution under exposure-matched conditions unresolved	Test sustained local exposure and immune-independent post-pulse injury without default priority
Suppressive myeloid-state change	TAMs/monocyte-derived myeloid cells/MDSCs	Compartment-specific active exposure and persistence of phenotypic or functional change; unresolved	Preclinical phenotype and function evidence for DHA and artesunate; distinct from MDSC abundance changes; not established under PK-matched conditions	Test depletion, recruitment, phenotype, transcription, and function separately; no default priority
Tumor stress–dendritic cell relay–CD8^+^ T-cell priming	Tumor cells plus dendritic cells	Tumor stress, antigen transport, and lymph-node priming after defined exposure; unresolved	Limited-to-moderate, particularly in combination settings; not demonstrated as a complete temporally ordered pathway under PK-matched conditions	Test temporal order, antigen-presentation competence, and causal necessity without default priority
PMN-MDSC/TAN barrier loosening	Granulocytic suppressor populations	Granulocytic exposure, functional suppression, and spatial access after a pulse; unresolved	Emerging neutrophil-like-cell evidence; *in vivo* barrier relief after matched exposure unresolved	Test barrier relief without assuming upstream placement
Direct CD8^+^ T-cell responsiveness	CD8^+^ T cells	Lymphocyte active exposure and changes in activation, survival, differentiation, or function; unresolved	Context-dependent artesunate activation and DHA-associated inhibition of activation markers; direct matched-pulse DHA effect unresolved	Test direct responsiveness and relay-mediated effects using the same causal criteria
Direct NK-cell responsiveness	NK cells	Lymphocyte active exposure and changes in activation, survival, differentiation, or function; unresolved	Limited	Test direct responsiveness and relay-mediated effects using the same causal criteria

PK compatibility refers to temporal plausibility under brief systemic exposure; plasma PK alone does not establish compartment-specific active exposure or rank mechanisms. None of the listed pathways is established as dominant under exposure-matched conditions.

## Experimental and translational implications for cancer immunology

The most informative next experiments will not be those that reconfirm tumor-cell suppression under continuous high-concentration DHA exposure. They will be those that compare tumor-intrinsic injury, direct immune-cell responses, and immune relay through their temporal and compartmental logic under exposure-matched conditions. The question goes beyond whether DHA can alter the tumor microenvironment. Which compartment receives the pharmacologic pulse first? Does the response persist after washout? Is it required for therapeutic benefit *in vivo*?

We propose four criteria for identifying leading *in vivo* mechanisms of DHA under route- and source-defined systemic exposure: compartment-specific exposure matching, temporal precedence, biological persistence after washout with residual active exposure assessed, and *in vivo* necessity. Confidence should rise only when all four are met—and in cancer immunology, each criterion must be examined by compartment, because a late rise in CD8^+^ T cells or NK cells reveals nothing about which compartment first received the pulse.

This sequence of events should also determine biomarker selection. If the lasting response depends on immune relay, early pharmacodynamic measurements should include tumor-intrinsic injury, direct lymphocyte responses, myeloid-state changes, dendritic-cell activation, granulocytic exclusion, chemokine networks, and antigen processing or presentation in the tumor and tumor-draining lymph node (20, 41, 43, 44). CD8^+^ T-cell priming would follow antigen presentation in the proposed relay, but early direct lymphocyte responses should also be assessed. Tumor-cell death alone would not adequately identify the initiating mechanism. Combination strategies should similarly favor treatments that can take advantage of improved antigen presentation, reduced myeloid suppression, or greater T-cell entry, particularly checkpoint blockade and other therapies requiring effective immune priming.

The proposed relay also requires a sufficiently responsive tumor immune microenvironment. This requirement is separate from the pharmacokinetic effects of delivery systems. In highly immune-excluded or immune-desert tumors, limited dendritic-cell and myeloid infiltration may prevent a transient initiating signal from being amplified (38). Severe immune escape can create a similar failure downstream. Defective antigen processing or MHC-I cross-presentation in dendritic cells may restrict priming, whereas loss of tumor-cell MHC-I or defects in tumor antigen processing may restrict CD8^+^ T-cell recognition despite successful priming; profound systemic T-cell exhaustion may further restrict effector activity (20, 44, 45). The model therefore predicts greater efficacy in tumors with some existing immune infiltration and preserved antigen-presentation machinery. Both conditions are experimentally assessable and should contribute to clinical biomarker stratification.

Finally, the existing clinical development literature makes the translational question more urgent rather than less so. A randomized placebo-controlled pilot study of oral artesunate in colorectal cancer and a phase I study of intravenous artesunate in advanced solid tumors both support the feasibility of clinical evaluation of artemisinin-based agents in oncology (8, 46). These studies alone do not validate the immune-relay model. However, they underscore a practical point: if measured DHA exposure following the specified oral or intravenous artesunate regimen is brief, then mechanistic interpretation, biomarker selection, and rational immunotherapy combinations should reflect this constraint from the outset. **Table 2** summarizes key experimental designs that compare pulse-triggered immune relay, persistent tumor-intrinsic injury, direct lymphocyte responses, and sustained drug action *in vivo*.

**Table 2:** Falsifiable immunologic predictions of the pulse-triggered immune relay model.

Experimental question	Suggested design	Result supporting pulse-triggered immune relay	Result weakening relay or supporting a competing mechanism
Is DHA efficacy immune-dependent?	Compare immunocompetent *and* immune-deficient models with matched plasma and tumor exposure	Loss of efficacy with host immunity disrupted; initiating compartment remains unresolved	Similar efficacy despite loss of host immunity
Are CD8^+^ T cells required for a major component of efficacy?	Anti-CD8 depletion or equivalent genetic/functional perturbation	Reduced effect establishes CD8^+^ T-cell contribution, not direct drug sensing or relay order	Little change weakens CD8 dependence but does not exclude other immune mechanisms
Are dendritic-cell relay pathways required?	Batf3 perturbation, cDC1 depletion, or functional blockade with tumor and draining-node antigen-presentation readouts	Reduced priming and diminished therapeutic efficacy	Minimal dependence on dendritic-cell pathways
Are suppressive myeloid compartments early receivers of the DHA pulse?	Early flow cytometry, scRNA-seq, spatial profiling, cell-loss and recruitment measurements, and functional assays after pulse-matched dosing	Early phenotypic or functional change precedes T-cell changes; abundance and transcription alone do not establish function	No early myeloid response weakens this initiating route but does not establish direct cytotoxicity
Does DHA relieve granulocytic immune exclusion?	Ly6G depletion, CXCR2-axis interrogation, and spatial T-cell access analysis	Greater effector-cell access linked to reduced granulocytic suppression	Unchanged exclusion weakens barrier relief, not immune mechanisms generally
Do biologic effects persist after washout?	Pulse-chase assays in tumor, myeloid, dendritic-cell, and purified lymphocyte systems with residual active exposure assessed	Persistent immune-relevant function after active exposure ends, linked to *in vivo* necessity	Persistent immune-independent tumor injury supports a competing post-pulse mechanism; loss after washout indicates exposure dependence
Does efficacy require retained active drug or persistent biological effects?	Measure paired plasma and tumor profiles, unbound and intracellular exposure, concentration–effect relationships, and post-pulse injury	No retained active exposure plus immune-pathway necessity supports relay; drug clearance alone does not	Retained active exposure with exposure-dependent killing, or persistent immune-independent injury after clearance, supports distinct direct mechanisms
Which compartment changes first *in vivo*?	Dense early time-course sampling across tumor and immune compartments	Tumor-stress/myeloid/DC changes precede lymphocyte expansion and are causally required	Early tumor injury may initiate direct effects or immune relay; early lymphocyte changes suggest a direct route if causally linked to efficacy

## Limitations, boundary conditions, and alternative explanations

The pulse-triggered immune relay model proposed here should be understood as a mechanism-prioritization framework, not as a universal explanation for all DHA-associated antitumor effects. Several boundary conditions could shift the balance between direct tumor-cell injury and immune-mediated relay.

First, plasma PK may misrepresent intratumoral exposure. Abnormal vascular permeability, tissue partitioning, stromal sequestration, local protein binding, cellular uptake, and slow washout may produce tumor exposure that differs from plasma-based estimates (47, 48). Accordingly, future studies should report paired plasma and tumor concentration–time profiles, tumor-to-plasma ratios, and, where technically feasible, unbound DHA concentrations in the tumor interstitium or relevant intracellular compartments. These measurements, linked to compartment-specific concentration–effect relationships, would help distinguish sustained local active exposure from persistent tumor-intrinsic injury and immune relay after exposure has ended.

Second, tumor-cell biochemistry can amplify even a brief DHA pulse. Because artemisinin derivatives can engage redox stress, heme/iron-dependent activation, and diverse tumor-intrinsic pathways (49, 50), tumors rich in labile iron, with active heme metabolism, high transferrin-receptor expression, mitochondrial fragility, or weak antioxidant defenses may continue to suffer damage long after the drug is cleared (51, 52). This interpretation remains consistent with a PK-constrained model but shifts the emphasis from sustained drug occupancy to persistent post-exposure cellular injury.

Third, even brief individual exposures can add up to cumulative pharmacodynamic remodeling when dosing is repeated. Intermittent DHA pulses may progressively alter tumor stress, myeloid-cell state, dendritic-cell activation, stromal barriers, or lymphocyte recruitment (53, 54). Studies should therefore distinguish single-pulse effects, repeated intermittent pulses with possible cumulative pharmacodynamic remodeling, continuous exposure, and sustained-release or local delivery, using both dosing schedules and measured concentration–time profiles.

Fourth, engineered delivery strategies, such as nanoparticle-based and sustained-release systems, can prolong local exposure, alter subcellular distribution, increase tumor selectivity, or intensify oxidative stress, thereby favoring mechanisms like direct cytotoxicity, ferroptosis-like injury, or immunogenic cell death (47, 55, 56). Until the exposure profile of such systems is re-measured, the present framework should not be applied.

Finally, immune relay and direct tumor-cell injury are not mutually exclusive. A brief DHA pulse may induce limited stress, partial death, or altered phagocytic visibility, which could be amplified by dendritic cells, myeloid-state change, and CD8^+^ T-cell priming (54, 57). The key question is not whether direct effects occur, but whether they suffice to explain durable *in vivo* efficacy without immune relay. The framework therefore compares prolonged local drug action, durable immune-independent tumor-cell injury, direct immune-cell responses, and immune relay under route-, formulation-, and exposure-matched conditions without assigning default mechanistic priority.

## Conclusion

What is needed now is a comparison of mechanisms consistent with DHA’s compartment-specific exposure patterns and the immune setting of cancer, rather than a default assignment to direct tumor-cell toxicity or immune relay. More broadly, DHA illustrates a recurring problem in cancer immunology: durable therapeutic effects may be mechanistically misassigned when short-lived pharmacologic inputs are interpreted through experimental systems built around continuous exposure. Resolving this issue will require joint testing of exposure matching, temporal sequence, persistence after washout, and *in vivo* necessity at the level of tumor and immune compartments. DHA may be better understood *in vivo* as a short pharmacologic input whose durable consequences can include both persistent tumor-intrinsic injury and amplification through the tumor immune microenvironment. The relative contributions and causal ordering of these components should be determined through exposure-matched, temporally resolved, compartment-specific, and perturbation-based studies.

## Data Availability

The original contributions presented in the study are included in the article/supplementary material. Further inquiries can be directed to the corresponding author.
